# RNA Splicing of the Abi1 Gene by MBNL1 contributes to macrophage‐like phenotype modulation of vascular smooth muscle cell during atherogenesis

**DOI:** 10.1111/cpr.13023

**Published:** 2021-03-23

**Authors:** Yinan Li, Xiangjiang Guo, Guanhua Xue, Han Wang, Yuli Wang, Weilun Wang, Shuofei Yang, Qihong Ni, Jiaquan Chen, Lei Lv, Yiping Zhao, Meng Ye, Lan Zhang

**Affiliations:** ^1^ Department of Vascular Surgery Renji Hospital Shanghai Jiao‐Tong University School of Medicine Shanghai China

**Keywords:** Abi1, alternative splicing, macrophage‐like vascular smooth muscle cells, MBNL1

## Abstract

**Background:**

Vascular smooth muscle cells (VSMC) switch to macrophage‐like cells after cholesterol loading, and this change may play an important role in atherogenesis. Muscleblind‐like splicing regulator 1 (MBNL1) is a well‐known splicing factor that has been implicated in many cellular processes. However, the role of MBNL1 in VSMC macrophage‐like transdifferentiation is largely unknown. In this study, we aim to characterize the role of MBNL1‐induced gene splicing during atherogenesis.

**Methods:**

The expression of MBNL1 and Abelson interactor 1 (Abi1) splice variants (Abi1‐e10 and Abi1‐Δe10) was compared between artery tissues from healthy donors and atherosclerosis patients. Regulatory mechanisms of MBNL1‐induced Abi1 gene splicing were studied, and the signal pathways mediated by Abi1 splice variants were investigated in VSMC.

**Results:**

Loss of MBNL1 was found in the macrophage‐like VSMC (VSMC‐M) in artery wall from atherosclerosis patients. In vitro and in vivo evidence confirmed that Abi1 is one of the MBNL1 target genes. Loss of MBNL1 significantly induces the Abi1‐Δe10 isoform expression. Compared to the known actin organization activities of the Abi1 gene, we discovered a novel action of Abi1‐Δe10, whereby Abi1‐Δe10 activates Rac1 independent of upstream stimulation and triggers the Rac1‐NOX1‐ROS pathway, which results in increased expression of transcription factor Kruppel‐like factor 4 (KLF4). While Abi1‐Δe10 inhibits contractile VSMC biomarkers expression and cell contraction, it stimulates VSMC proliferation, migration and macrophage‐like transdifferentiation.

**Conclusion:**

Loss‐of‐function of MBNL1 activates VSMC‐M transdifferentiation to promote atherogenesis through regulating Abi1 RNA splicing.

## Graphical abstract

Loss of MBNL1 significantly induces the Abi1‐Δe10 isoform expression by alternative splicing in the macrophage‐like VSMC (VSMC‐M) in artery wall from atherosclerosis patients. A novel action of Abi1‐Δe10, whereby Abi1‐Δe10 actives Rac1 independent of upstream stimulation, was discovered. Abi1‐Δe10 triggers the Rac1‐NOX1‐ROS pathway to upregulate KLF4, which results in inhibition of contractile VSMC biomarkers expression and cell contraction and stimulation VSMC proliferation, migration and macrophage‐like transdifferentiation.

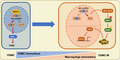

## 1 BACKGROUND

Atherosclerosis is a chronic inflammatory condition that results from complex interactions of modified lipoproteins and various cell types of the vessel wall.^1^ The detail that each particular cell type contributes to atherogenesis is not completely understood. Vascular smooth muscle cells (VSMC) are the main cell type of vascular wall and a major component of atherosclerosis.^1^ It is generally accepted that VSMC is not terminally differentiated and can undergo phenotypic transdifferentiation during atherosclerosis development.^1^, ^2^, ^3^ Accumulating evidence has suggested that VSMC can convert to macrophage‐like cells that have lost classical contractile ability and make up a major component of atherosclerotic lesions.^2^, ^3^, ^4^, ^5^ VSMC‐derived macrophage‐like cell (VSMC‐M) expresses typical macrophagic biomarkers such as CD68 but low level of contractile VSMC biomarkers αSMA. Unlike contractile VSMC, VSMC‐M is highly proliferative and exerts enhanced macropinocytosis function which engaged cholesterol accumulation and foam cell expansion.^4^ That these phenomena are clinically relevant is supported by studies showing the presence of cells expressing both VSMC and macrophage biomarkers in human atherosclerotic plaques, highlighting the importance of VSMC‐M transdifferentiation in atherosclerosis.^2^, ^6^ Several mechanisms about VSMC‐M transdifferentiation have been purposed; however, knowledge remains to be elucidated.

The development of VSMC‐M is regulated by cell lineage plasticity, whereby VSMC cells acquire a stem‐like phenotype followed by dedifferentiation to VSMC‐M.^7^, ^8^ The most well‐defined core ‘stemness’ genes in human cells are OCT4, SOX2, c‐Myc and KLF4 which are powerful enough to reprogram terminally differentiated cells into induced pluripotent stem cells.^9^ It has been reported that KLF4 is not expressed in differentiated VSMC, but can be induced by oxidized phospholipids and inflammatory cytokines, downregulating the expression of VSMC contractile biomarkers while inducing expression of macrophage biomarkers.^8^, ^10^ Apoe−/− mice with VSMC‐specific conditional knockout of KLF4 had a decreased number of VSMC‐M cells.^11^ These findings suggest that upregulation KLF4 may regulate a stem‐like gene network to confer VSMC cell lineage plasticity for VSMC‐M development.

Abelson interactor 1 (Abi1) is a key component of several intrinsic complexes that regulate actin cytoskeletal remodelling upon upstream stimulation.^12^, ^13^, ^14^ Alteration of Abi1 has been reported to associate with cell proliferation, migration and macropinocytosis.^15^, ^16^, ^17^ Through alternative splicing, numerous structurally distinct variants of Abi1 exist in human cells,^17^, ^18^ providing a potential diversity of Abi1 regulatory signalling, suggesting the possible existence of potential mechanisms through which Abi1 regulates VSMC‐M development.

Recently research has focused on identifying the genomic and transcriptomic alteration profile of VSMC‐M, and studies of alternative splicing have lagged. Through analysing RNA‐seq data, we found that an Abi1 splicing variant that lacking exon10 (Abi1‐Δe10) is upregulated in VSMC‐M. Abi1‐Δe10 splicing is downregulated by splicing factor MBNL1 which expression was decreased in VSMC‐M. This splicing event activates Rac1 in absence of upstream stimulating and promotes KLF4 expression through a NADPH oxidase 1 (NOX1)‐dependent pathway. Our research provides a novel insight into the splicing regulatory mechanism in VSMC phenotype modulation during atherogenesis.

## 2 MATERIALS AND METHODS

### 2.1 Tissue collection

Artery samples were collected from peripheral artery atherosclerosis patients who received thigh amputation, and control arteries were obtained from healthy people who underwent amputation by accidents.^19^ Demographic information of the patients was provided in Supplemental Table S1. Vascular segments with atherosclerotic lesions and control tissues were collected for further analysis. All specimens were obtained from Renji Hospital, School of Medicine, Shanghai Jiaotong University, and were processed in the Biobank of Renji Hospital. All procedures were approved by the research ethics committee of Renji Hospital (RA‐2020‐071).

### 2.2 Immunofluorescence assays

Tissues were fixed in 4% paraformaldehyde, permeabilized in 0.25% Triton X‐100 and blocked with 1% BSA for 1 hour at room temperature. Cells were then incubated with the primary antibody, washed with PBS with 0.1% Triton X‐100 and incubated with FITC‐conjugated secondary antibody (1:1000 in PBST containing 1% BSA). Tissue imaging was captured by Zeiss fluorescent microscope (Carl Zeiss).

### 2.3 RNA in situ hybridization analyses

An Abi1‐e10‐specific RISH probe targeting the bp of Abi1‐e10 mRNA, an Abi1‐Δe10‐specific RISH probe targeting the bp of Abi1‐Δe10 mRNA and a negative control probe (targeting the dapB gene from bacteria) were designed. RISH assays were performed by using the QuantiGene ViewRNA ISH Assay Kit (Panomics) following the manufacture's protocol.

### 2.4 Flow cytometry

Tissues were digested by collagenase and cells were suspended with 1X TrypLE Express (Gibco) individually, blocked in PBS (Sigma) containing 0.1% FBS (Gibco) and stained with fluorescent antibodies against CD68 or CD45. PBS‐treated cells served as a control.

### 2.5 Cell lines and transfection

Human primary aortic smooth muscle cells were purchased from American Type Medium (ATCC PCS‐100‐030) with Vascular Smooth Muscle Cell Growth Kit (ATCC PCS‐100‐04). Lipofectamine 3000 (Invitrogen) and SuperFect Transfection Reagents (Qiagen) were used for plasmid and siRNA transfection.

### 2.6 PCR and Immunoblotting assays

Real‐time qPCR assays were performed as described.^19^ All real‐time qPCR assays were carried out using three technical replicates and three independent cDNA syntheses. Primer information was listed in Supplementary Table S2. Western blotting assays were performed as reported.^19^ For nuclear fraction assay, cytosol and nuclear protein lyse were extracted using NE‐PER nuclear and cytoplasmic extraction kit (Life Technology). Antibody information was listed in Supplementary Table S3. Experiments were repeated in three independent experiments, and one of the representative blots was shown.

### 2.7 Cell proliferation and migration assays

Cell proliferation and migration assays were described previously.^19^, ^20^ Briefly, the cell proliferation assay was performed using the MTS (Promega) reagent according to the manufacture's protocol. Cell proliferation rates were calculated as relative fold change of OD450. For migration assays, a monolayer wound was created when cells reached 100% confluence. Cell migration was subsequently captured at time point 0 and 24 hours after wound scratch. The migration ability of cells was calculated as the migration distance from 0h to 24 hours.

### 2.8 Co‐immunoprecipitation

Whole‐cell lysates were extracted by the buffer containing 50 mM of Tris pH8.0, 150 mM of NaCl, 1% NP40, 0.5% sodium deoxycholate, 0.1% SDS, and proteinase and phosphatase inhibitors (Roche). Immunoblotting assays follow the standard protocol as reported.^20^ Information on antibodies is listed in materials. When performing co‐IP assays, cell lysates were extracted by NETN buffer containing 0.5% NP40, 1 mM of EDTA, 50mM of Tris, and 150mM of NaCl plus proteinase and phosphatase inhibitor (Roche). Pre‐cleared lysates were incubated with indicated antibodies, and the associated proteins were immunoblotted by antibodies as indicated. Experiments were repeated at least three times, and one set of the representative blots was shown.

### 2.9 Abi1‐minigene construction and in vivo splicing assays

The human genomic BAC clone (RP11‐75E16) was used as the template for PCR to amplify exon9, exon10, exon11, and their flanking intron regions (~300‐400 base pairs) of Abi1 gene (NM_001012751) by Platinum Taq DNA Polymerase High Fidelity (Invitrogen). The Abi1‐minigene with mutant was generated using the original Abi1‐minigene as the template. The integrity of the final construct was confirmed by DNA sequence.

### 2.10 RNA pulldown assay

RNA pulldown assay was performed as previously described.^20^ VSMC cells transfected with Flag‐MBNL1 plasmid were lysed in NETN buffer (0.5% NP40, 1 mM EDTA, 50 mM Tris and 150 mM NaCl with proteinase inhibitor). 0.4 nmol biotin‐labelled RNA oligonucleotides (Invitrogen) were banded onto 100 μL of streptavidin beads (Pierce) in a final volume of 500 μL of binding buffer DG (20 mM HEPES‐KOH, pH 7.9, 80 mM potassium glutamate, 0.1 mM EDTA, 1 mM DTT and 20% glycerol) at 4°C for 2 hours. The Flag‐MBNL1 protein was purified by incubating cell lysate with the beads containing biotin‐labelled RNA oligonucleotides at 4°C for 2 hours. Then the beads were washed with binding buffer DG and suspended in SDS loading buffer. Eluted proteins were analysed by Western blot and detected by Flag‐tag antibody.

### 2.11 RNA immunoprecipitation assays

RNA immunoprecipitation assay was performed as previous described.^20^ Total RNA of VSMC cell transfected with Flag‐MBNL1 plasmid was cross‐linked with formaldehyde (10 mL PBS + 270 μL 1% formaldehyde) for 20 minutes at 37°C and sonicated in 300 μL buffer I (1% SDS, 10 mM EDTA, and 50 mM Tris, pH 8.0, plus protease inhibitor cocktail). After centrifugation, the supernatants were added to 2.7ml buffer II, and 30 μL of the mixture was saved as input. For immunoprecipitation, 2 μg of Flag antibody or control IgG antibody was added to the purified chromatin sample and incubated overnight at 4°C. Immunocomplexes were precipitated by adding 50 μL of protein A/G agarose beads for 2 hours at 4°C with agitation. Beads were washed sequentially for 5 minutes each in 1 mL of buffers III‐VI, as described previously.^20^ Immunocomplexes were eluted by adding 1600 μL of elution buffer (1% SDS and 0.1 M NaHCO3) and 50 μL RNase inhibitor to beads. 500 μL of eluted immunocomplexes was added in 10 μL 5 M NaCl and subsequently heated for 2 hours at 64°C to reverse formaldehyde‐induced cross‐links. RNA segments were isolated and collected by 1.5 mL lysis buffer with 15 μL 2‐mercaptoenthanol using Purelink RNA Mini Kit (Ambion) according to the manufacture's instruction and subsequence to reverse transcription and analysed by real‐time qPCR as described above. Data were calculated as a percentage of input.

### 2.12 Lentivirus production, infection and generation of stable cell lines

To generate VSMC lines stably expressing Abi1‐e10 and1Abi1‐Δe10, the Abi1‐e10 and Abi1‐Δe10 cDNA were cloned into the FU(GW)BW‐blasticidin‐resistant lentivirus vector (purchased from Addgene) using Gateway Technology (Invitrogen) according to the manufacture's instruction. 293T packaging cells were co‐transfected with viral vectors, VSV‐G‐encoding plasmid and pCMV R8.9. The supernatants of transfected 293T cells containing viruses were used to infect VSMC cells in the presence of 10%FBS. The infected LNCaP cells were selected in the presence of 5μg/ml blasticidin. Expression of Abi1‐e10 and Abi1‐Δe10 was confirmed by both Western blot and real‐time qPCR.

### 2.13 Small GTPase Rac1 activity pull‐down assay

Rac1 activity was measured by GTP pull‐down assay. Briefly, VSMC were lysed with buffer containing 50 mmol/L of Tris‐HCl (pH 7.40, 1 mM EDTA, 150 mM NaCl, 1 mM phenylmethylsulphonyl fluoride, 1% Triton X‐100, 1 mM sodium fluoride and proteinase inhibitor cocktail tablet). Lysates were incubated with glutathione S‐transferase/p21‐activated protein kinase/Rac interactive binding domain (GST‐PAK‐CRIB) and glutathione Sepharose‐4B beads (Thermo Fisher Scientific). Beads were then briefly washed. Bound proteins were eluted by boiling in 2× SDS sample buffer and then subjected to 12% SDS‐PAGE followed by immunoblotting with the Rac1 antibody. Blots were analysed with densitometry.

### 2.14 ROS detection

In cultured VSMC, the ROS levels were determined immediately after sample collection. Cellular ROS levels were assessed by measuring CM‐H2DCFDA (Thermo Fisher) fluorescence. All values were normalized to VSMC protein concentration.

### 2.15 Statistics

Statistical analysis was carried out by the GraphPad Prism 8.0 software. Differences among groups were compared by Student's *t* test. The level of significance was set at *P* <.05 as *, *P* <.01 as **.

## 3 RESULTS

### 3.1 RNA splicing of Abi1‐Δe10 is upregulated in macrophage‐like VSMC‐derived from atherosclerotic tissue

**FIGURE 1:**
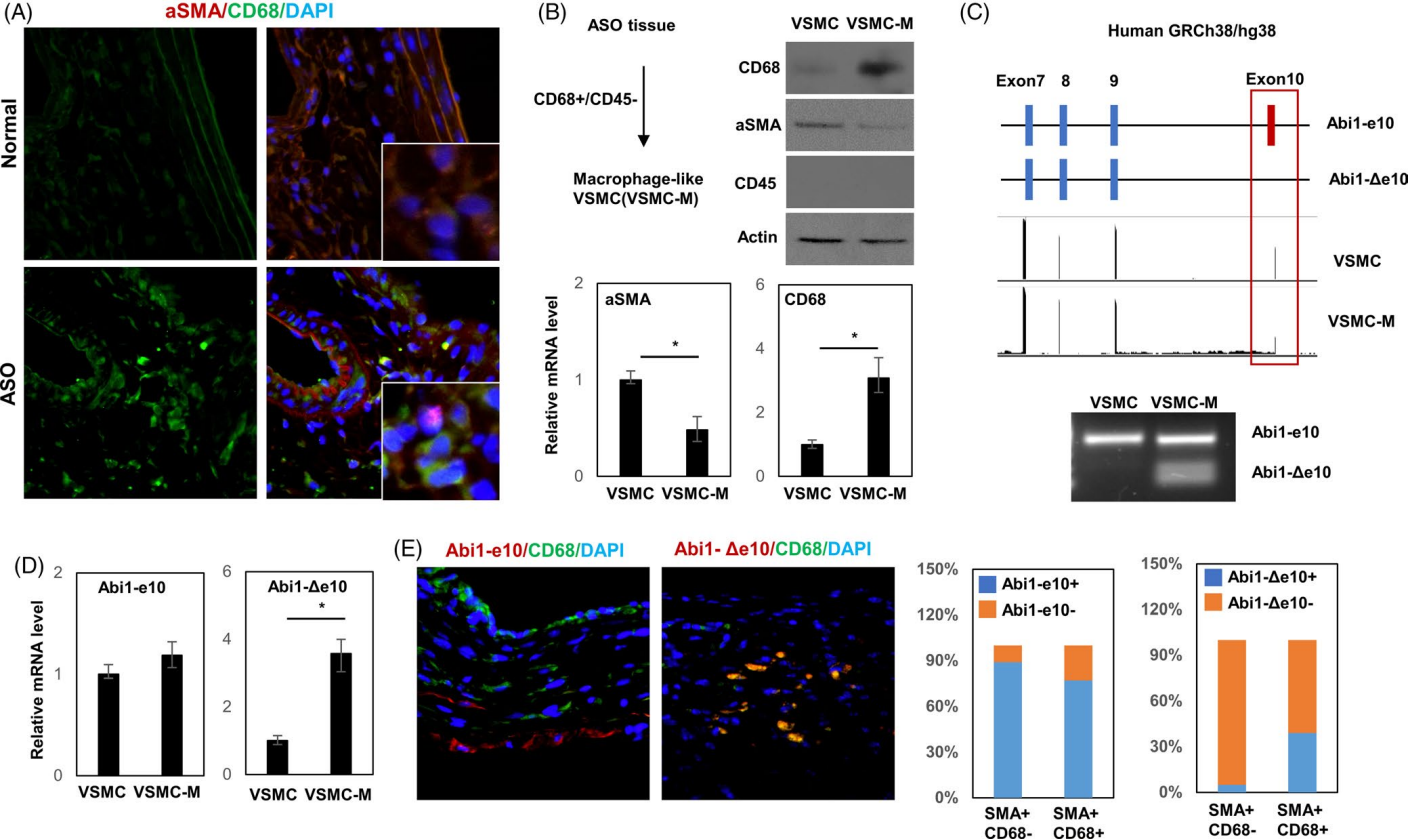
Abi1 gene splicing is increased in macrophage‐like VSMC derived from ASO neointima. (A) Immunofluorescence staining for α‐SMA (red, Alexa Fluor 555) and CD68 (green, Alexa Fluor 633) on arteries isolated from patients with severe ASO. Arteries from healthy donors were also stained as control. Representative images of co‐staining were shown. (B) αSMA+/CD68+/CD45‐ (VSMC) and αSMA+/CD68‐/CD45‐ (VSMC‐M) cells were selected by flow cytometry and validated by immunoblotting and real‐time qPCR. (C) RNA‐Seq reads from VSMC and VSMC‐M were mapped with Star using the Ensembl gene annotations GRCh38.87. Decreased inclusion of the alternatively spliced exon10 of Abi1 gene in VSMC‐M was identified and validated by RT‐PCR. (D) Expression of Abi1‐e10 and Abi‐Δe10 was determined by real‐time qPCR. (E) RISH staining for CD68 and Abi1‐e10/Abi1‐Δe10 on the smooth muscle of artery tissue isolated from patients with severe ASO and healthy donors. Representative images of co‐staining were shown. The distribution of Abi1‐e10 and Abi1‐Δe10 mRNA indifferent group was plotted

It has been reported that vascular smooth muscle cells (VSMC) will transdifferentiate into a macrophage‐like phenotype (VSMC‐M) during atherogenesis. To identify the phenotype of VSMC with the use of cell‐specific markers, we first isolated the intima of control and atherosclerotic artery as described in the Material and Methods section. By double immunofluorescence staining for macrophage biomarker CD68 and smooth muscle biomarker αSMA, we found that the amount of CD68+ VSMC was significantly increased in the thickened intima of ASO arteries (Figure 1A). To evaluate the origin of these CD68+ cells, we also applied double immunofluorescence staining for CD68 and myeloid biomarker CD45. Among tissue cells in the intima of ASO arteries, about 50% of them were CD68+/CD45−, but only 20.8% were CD68+/CD45+, indicating that the majority of CD68+ cells were not originated from monocyte (Figure S1). These results demonstrated the upregulation of VSMC‐M in the hyperplastic intima. We further isolated and selected VSMC‐M by flow cytometry and validated using real‐time qPCR and immunoblotting (Figure 1B). RNA‐seq data comparing VSMC and VSMC‐M indicated that reads of Abi1 exon10 were high in VSMC but significantly decreased in VSMC‐M, indicating an Abi1 isoform lacking exon10 (Abi1‐Δe10) was induced during VSMC phenotype modulation (Figure 1C). To validate these findings in vitro and in vivo, we first analysed Abi1‐e10 and Abi1‐Δe10 mRNA levels in VSMC and VSMC‐M. We confirmed that Abi1‐Δe10 mRNA was strongly expressed in VSMC‐M; meanwhile, Abi1‐e10 expression showed no difference (Figure 1D). Next, we analysed the co‐expression of Abi1‐e10 and Abi1‐Δe10 mRNA with αSMA and CD68 by RNA in situ hybridization (RISH) assays. Only 5% CD68‐ VSMC showed positive Abi1‐Δe10 signalling. In contrast, up to 39% CD68 + VSMC were Abi1‐Δe10 positive. (Figure 1E). These data suggest that elevated Abi1‐Δe10 expression may correlate with the VSMC transdifferentiation and development of ASO.

### 3.2 Loss of MBNL1‐induced RNA splicing of Abi1‐Δe10 in VSMC macrophage‐like VSMC

**FIGURE 2:**
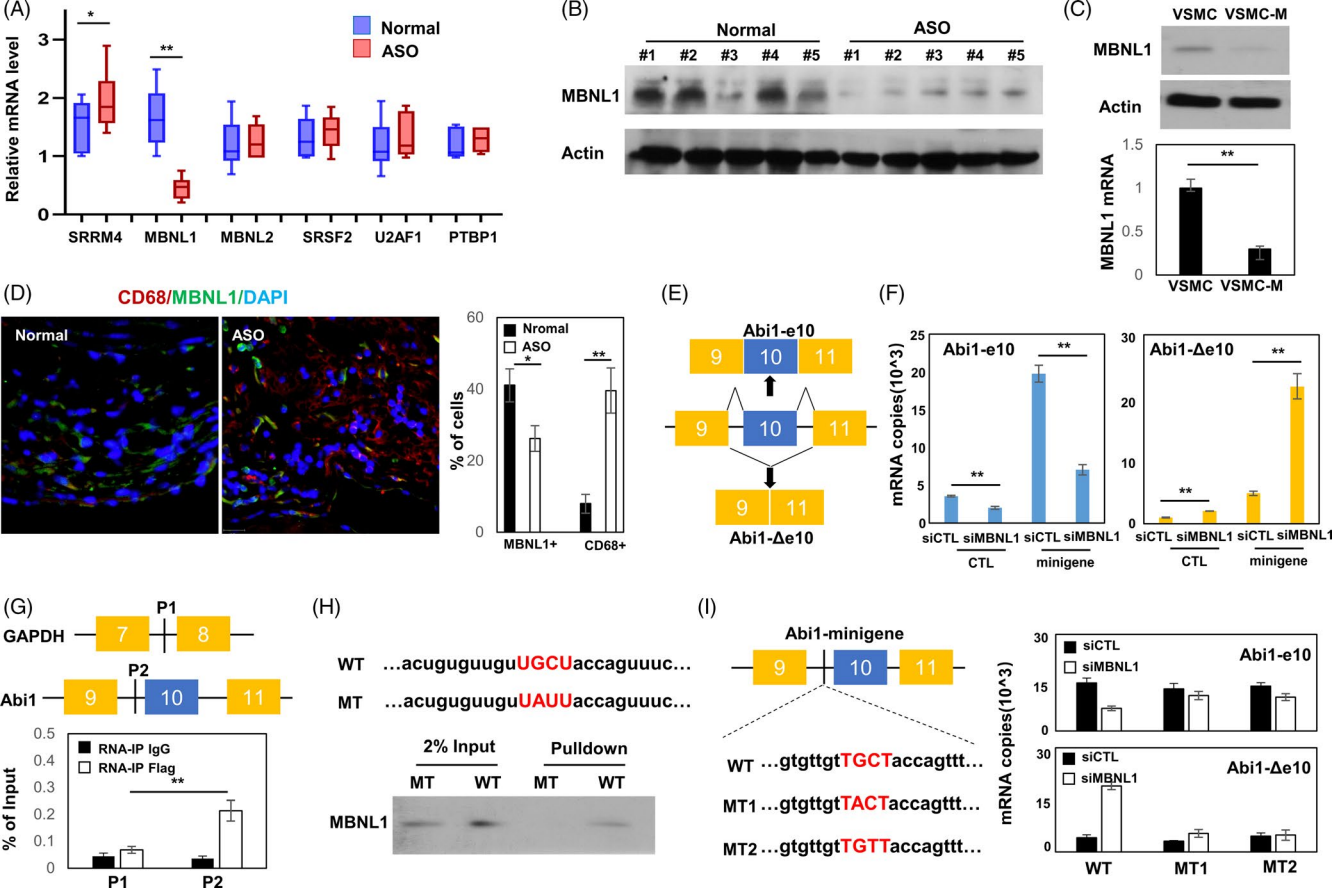
MBNL1 regulates RNA splicing of Abi1‐Δe10 macrophage‐like VSMC. (A) Total RNA of ASO and control intima was isolated to measure mRNA level of different splicing factors by real‐time qPCR. (B) Protein lyses were extracted from ASO and control intima, and immunoblotted with MBNL1 and actin antibodies. (C) VSMC and VSMC‐M were used to measure MBNL1 mRNA and protein expression by real‐time qPCR and immunoblotting. (D) ASO and control intima were co‐stained for MBNL1 and CD68. Representative images of co‐staining were shown. (E) A schematic diagram shows the Abi1 minigene reporter and the derived splice variants. (F) The Abi1‐minigene reporter was co‐transfected with siRNA targeting MBNL1 in VSMC cells. Total RNA was extracted to measure Abi1‐e10 and Abi1‐Δe10 mRNA levels by real‐time qPCR. (G) A schematic diagram shows the P1 and P2 regions used in in vivo RNA binding assays. RNA protein complexes in VSMC cells were cross‐linked by formaldehyde and immunoprecipitated with control or Flag antibody. Eluted RNA fragments were used as templates for real‐time qPCR to amplify the P1 and P2 regions. Signals were calculated as the percentage of input. (H) Biotin‐labelled RNA oligos containing wild‐type and mutant UGCU motifs were incubated with streptavidin‐conjugated beads. They were used to pull down protein extracts from VSMC cells. Proteins associated with streptavidin beads were eluted and immunoblotted with the MBNL1 antibody. (I) VSMC cells were transfected with control, Abi1‐minigene or Abi1‐minigene with MT1/MT2 mutations in the presence of ∓ siMBNL1 RNA. Total RNA was collected and used to measure Abi1‐e10/Abi1‐Δe10 mRNA levels by real‐time qPCR. All immunoblotting, real‐time qPCR and RNA binding assays were repeated in three independent experiments that were performed in triplicate. Data were presented as the mean ± SD ** delegates *P* <.01 when comparing with controls. One‐way ANOVA followed by Tukey test was used in pairwise comparison among different groups

Splicing of Abi1 has been reported to be associated with several splicing factors. We profiled the mRNA level of these factors and found that the change of MBNL1 mRNA level was the most significant between control and atherosclerosis arteries (Figure 2A). This result was validated by immunoblotting showed that MBNL1 protein was downregulated in ASO intima (Figure 2B). These findings were consistent with both MNBL1 mRNA and protein expression was dramatically decreased in VSMC‐M (Figure 2C). In vivo immunofluorescences results showed decreased MBNL1 level was accompanied with increased CD68 level (Figure 2D). Together, these results establish a correlation of MBNL1 suppression with Abi1‐Δe10 splicing.

To confirm that MBNL1 regulates Abi1 splicing, we constructed an Abi1 minigene reporter in which exon10 and is flanking ~ 300bp nucleotides were inserted between exons 9 and 11 and transfected into VSMC. MBNL1 depletion upregulated Abi1‐Δe10 but downregulated Abi1‐e10 mRNA derived from the minigene reporter (Figure 2E‐F). In vivo RNA binding assays showed that MBNL1 was recruited to the region next to the 3’ splice site of Abi1 intron 9 (P1 region), but not the control P2 region (Figure 2G). The UGCU motif was predicted to be a consensus MBNL1 recognition site and RNA pulldown assays confirmed that MNBL1 protein from VSMC interacted with the wild type, but not the mutant UGCU motif within the intron 9 (Figure 2H). Site‐directed mutagenesis (UGCU to UAUU) within the Abi1 minigene showed a failure of MBNL1‐mediated exon10 inclusion (Figure 2I). These results confirmed that MBNL1 regulates Abi1‐Δe10 splicing.

### 3.3 Loss of MBNL1 promotes phenotype modulation of VSMC through upregulating Abi1‐Δe10 isoform

**FIGURE 3:**
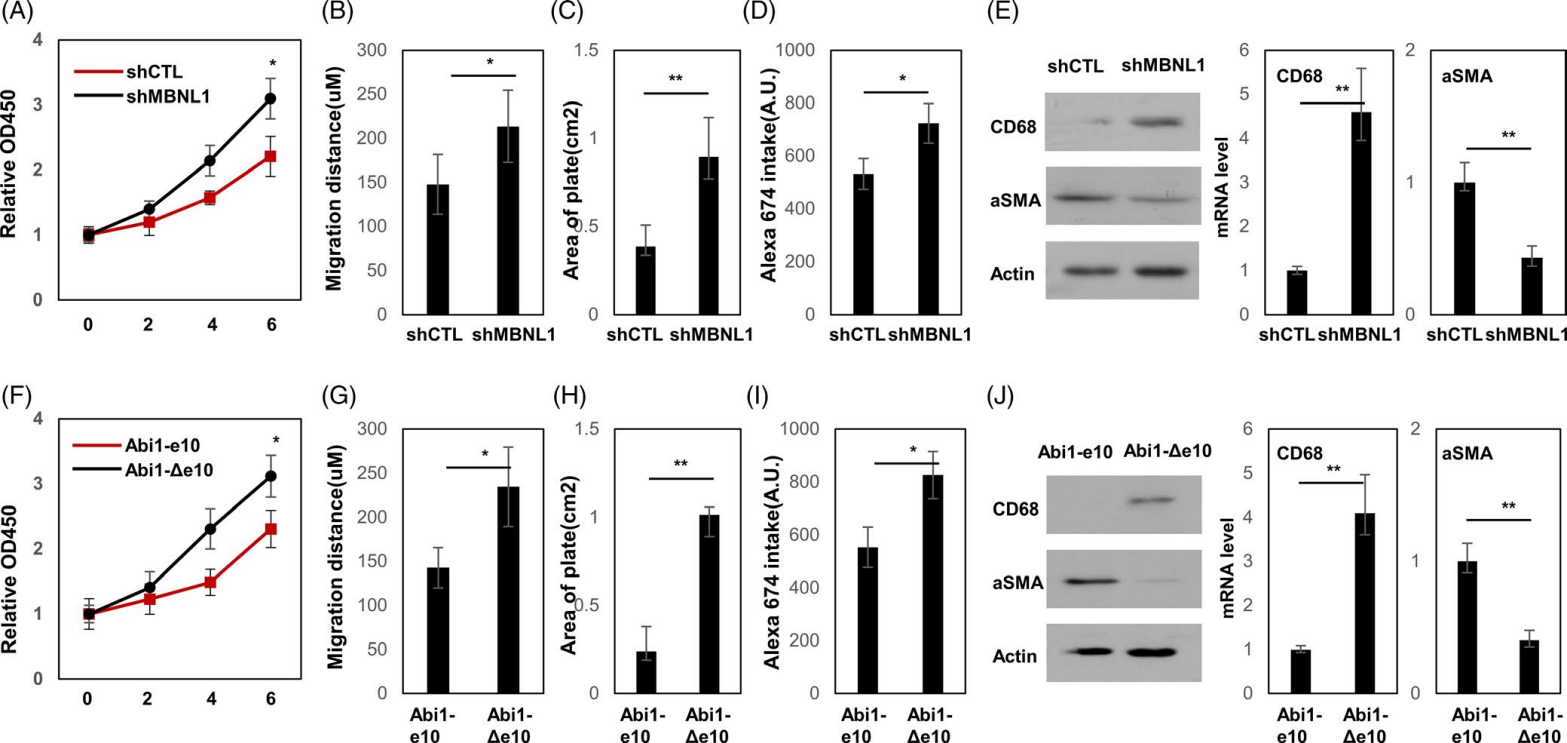
Abi1‐Δe10 enhances VSMC macrophage‐like characters. (A‐E) VSMC cell lines were stably transduced by control and shMBNL1 lentivirus. (F‐J) VSMC cell lines were stably transduced by Abi1‐e10 and Abi1‐Δe10 lentivirus. (A and F) Cell proliferation rates were measured by MTS assays and presented as relative fold change to day 0. (B and G) Wound healing assays were performed as described in the Methods section to measure VSMC cell migration rates within 16 hours. Cell images were captured by Zeiss fluorescent microscope and migration distances between 0h to 16h time points were measured using the ImageJ software. (C and H) Cell contraction assays were performed as described in the Methods section to measure VSMC cell contraction ability within 16 hours. Cell images were captured by Zeiss fluorescent microscope, and the area of the contraction plates was measured using the ImageJ software. (D and I) Uptake of Alexa Fluor 647 assay was performed in VSMC cell. Quantification of intracellular accumulation of Alexa Fluor 647 following a 2‐hour incubation with the dye was plotted. (E and J) The mRNA and protein expression of CD68 and aSMA were measured by real‐time qPCR and immunoblotting

Because MBNL1 is strongly expressed in VSMC but barely expressed in VSMC‐M, we introduced shRNA targeting MBNL1 into VSMC by lentivirus to study functions of MBNL1 (Figure S2A‐B). MBNL1 depletion significantly increased proliferation and migration rate and decreased contraction ability of VSMC (Figure 3A‐C). VSMC‐M is characterized as enhanced macropinocytosis and increased macrophage biomarkers expression. A fluorescent, water‐soluble dye (Alexa Fluor 647) was added into the culture medium as the macropinocytic target. As demonstrated in Figure 3D, loss of MBNL1 significantly increased uptake of the fluorescent dye, indicating enhanced macropinocytosis upon MBNL1 depletion. MBNL1 depletion also reduced αSMA expression and upregulated CD68 and pro‐inflammation genes TNFα and CCL2 expression (Figure 3E and Figure S3A). These results demonstrated that loss of MBNL1 engages macrophage transdifferentiation of VSMC. Since Abi1 is the target gene of MBNL1, we sought to determine whether the different Abi1 isoforms differentially regulated macrophage transdifferentiation in VSMC stably expressing Abi1 isoforms (FigureS2C‐D). It is Abi1‐Δe10 but not Abi1‐e10 stimulated VSMC proliferation, migration, but inhibited contraction (Figure 3F‐H). VSMC(Abi1‐Δe10) showed increased macropinocytosis determined by higher dye uptake (Figure 3I). Upregulation of CD68, TNFα and CCL2 accompanied with downregulation of αSMA was also observed in VSMC(Abi1‐Δe10) (Figure 3J & Fig. S3B). Collectively, these results indicate that loss of MBNL1 conferred VSMC macrophage‐like phenotype by alternative splicing of Abi1‐Δe10.

### 3.4 Abi1‐Δe10 actives the Rac1 signalling

**FIGURE 4:**
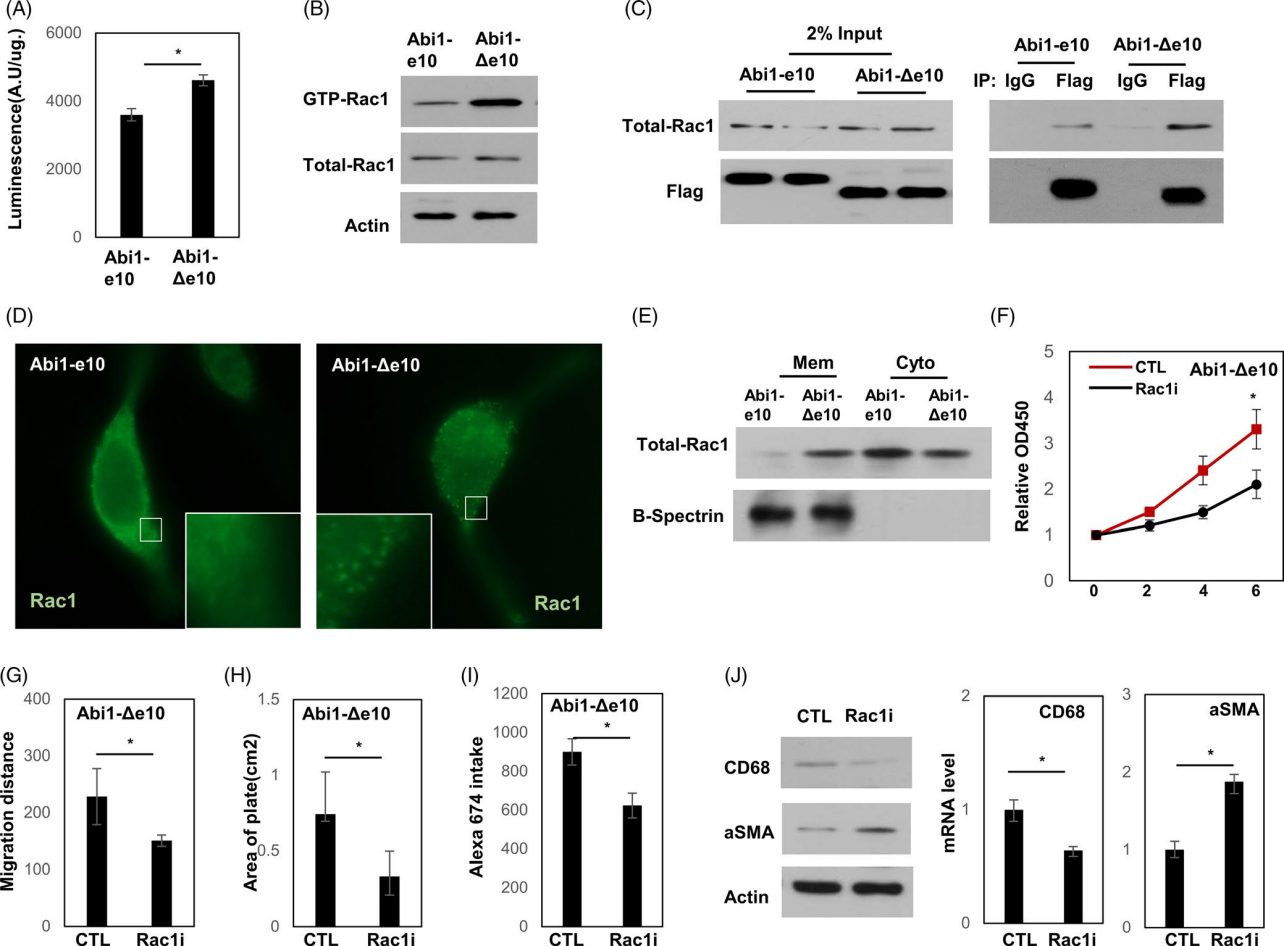
Abi1‐Δe10 promotes Rac1 activation. (A) Rac1 activity was evaluated by luminescence assay in VSMC(Abi1‐e10) and VSMC(Abi1‐Δe10) cells as described in the Methods section. (B) Activation of the GTPases Rac1 and total Rac1were determined in VSMC(Abi1‐e10) and VSMC(Abi1‐Δe10) cells as described in the Methods section. (C) VSMC(Abi1‐e10) and VSMC(Abi1‐Δe10) cells were used to perform immunoprecipitation assays with the Flag‐tag antibody. The associated proteins were detected by Rac1 and Flag antibodies. (D) VSMC(Abi1‐e10) and VSMC(Abi1‐Δe10) cells were used to perform immunofluorescence assays with Rac1 antibody. (E) Membrane fractions of VSMC(Abi1‐e10) and VSMC(Abi1‐Δe10) protein lysis were extracted. B‐Spectrinand was detected by immunoblotting and used as the marker to confirm the efficacy of membrane fraction. (F‐J) VSMC(Abi1‐Δe10) cell line was cultured with control or Rac1 inhibitor CAS 1177865‐17‐6 (Rac1i) (F) Cell proliferation rates were measured by MTS assays. (G) Wound healing assays were performed to measure VSMC cell migration rates within 16 hours. (H) Cell contraction assays were performed to measure VSMC cell contraction ability within 16 hours. (I) Uptake of Alexa Fluor 647 assay was performed in VSMC cell. Quantification of intracellular accumulation of Alexa Fluor 647 following a 2‐hour incubation with the dye was plotted. (J) The mRNA and protein expression of CD68 and aSMA were measured by real‐time qPCR and immunoblotting

Rac1 and Abi1 are components of the WAVE2 complex. To understand further the role of Rac1 and Abi1, we examined levels of activated Rac1 in VSMC expression different Abi1 isoforms. Both G‐LISA Rac1 activation assay and PAK‐CRIB pulldown assay showed that the VSMC(Abi1‐e10) had lower levels of activated Rac1 than did the VSMC(Abi1‐Δe10) (Figure 4A‐B). Co‐IP assays indicated that Abi1‐Δe10 had a higher affinity to Rac1 than Abi1‐e10 (Figure 4C). Rac1 is known to localize to the actin‐based membrane to exert its function upon upstream stimulation. We applied immunofluorescence microscopy to show that Rac1 was only localized in cytoplasm in VSMC(Abi1‐e10), while translocated into the membrane in VSMC(Abi1‐Δe10) (Figure 4D). These results were confirmed by immunoblotting assays using cytoplasm and membrane fractions of VSMC cells transfected with Abi1‐e10 and Abi1‐Δe10 (Figure 4E). These results indicated that Abi1‐Δe10 may bypass upstream stimulation to activate Rac1 and its downstream signalling. Consistently, Rac1 inhibition to VSMC(Abi1‐Δe10) cells caused a reduction in cell proliferation and migration but an increase in cell contraction (Figure 4F‐H). Furthermore, this treatment to VSMC(Abi1‐Δe10) also suppressed cell macropinocytosis and reversed Abi1‐Δe10‐induced αSMA and CD68 alteration (Figure 4I‐J). Taken together, these results reveal a new function of Abi1‐Δe10 that activating the Rac1 signalling to promote VSMC macrophage‐like transdifferentiation.

### 3.5 Abi1‐Δe10 activates the Rac1‐NOX1‐ROS signalling to active KLF4

**FIGURE 5:**
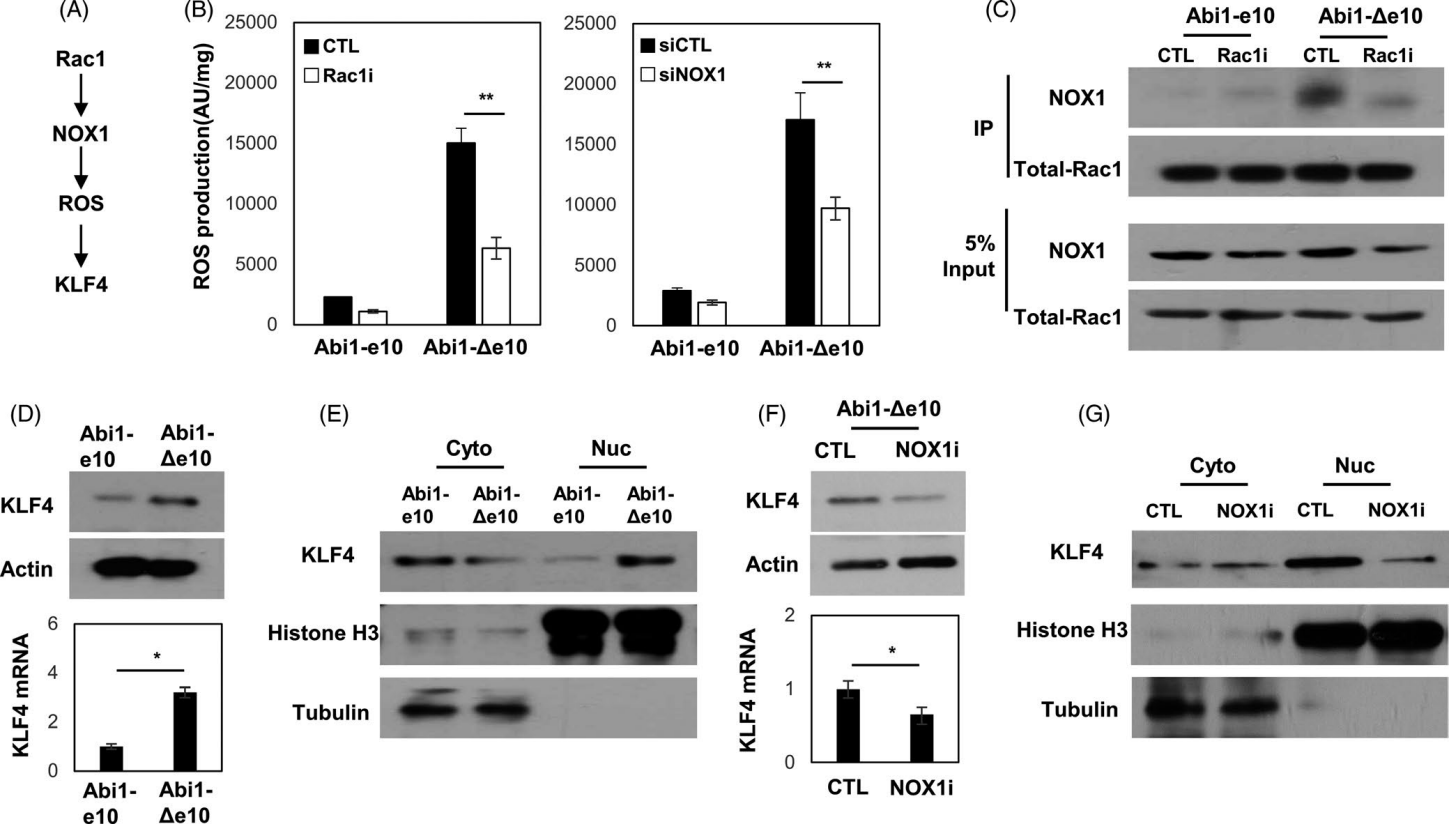
Abi1‐Δe10 triggers the Rac1‐Nox1‐KLF4 pathway. (A) A schematic diagram shows Rac1 regulation of the activity of KLF4 by the Rac1‐NOX1‐ROS pathway. (B) ROS production was evaluated in VSMC(Abi1‐Δe10) cells in the presence of ∓ Rac1 or siNOX1 RNA as described in the Methods section. (C) VSMC(Abi1‐e10) and VSMC(Abi1‐Δe10) cells in the presence of ∓ Rac1 were used to perform immunoprecipitation assays with the Rac1 antibody. The associated proteins were detected by Rac1 and NOX1 antibodies. (D) The mRNA and protein expression of KLF4 were measured by real‐time qPCR and immunoblotting. (E) Cytoplasmic and nuclear fractions of protein lysis of VSMC(Abi1‐e10) and VSMC(Abi1‐Δe10) cells were extracted. Histone H3 (H3) and tubulin were detected by immunoblotting and used as markers to confirm the efficacy of cytosol and nuclear fraction. The protein expression of KLF4 was measured by immunoblotting. (F) The mRNA and protein expression of KLF4 in VSMC(Abi1‐Δe10) cells in the presence of ∓ NOX1 inhibitor Apocynin (NOX1i) were measured by real‐time qPCR and immunoblotting. (G) Cytoplasmic and nuclear fractions of protein lysis of VSMC(Abi1‐Δe10) cells in the presence of ∓ NOX1i were extracted. Histone H3 (H3) and tubulin were detected by immunoblotting and used as markers to confirm the efficacy of cytosol and nuclear fraction. The protein expression of KLF4 was measured by immunoblotting

Recent evidence extends VSMC plasticity to transdifferentiation into macrophage‐like cells during atherogenesis that is dependent on KLF4, a transcription factor that induces cell dedifferentiation. Rac1 may promote VSMC transdifferentiation through modulating the expression and function of KLF4. Specifically, Rac1 activates the NADPH oxidase catalytic subunits 1 (NOX1) which in turn increases reactive oxygen species (ROS) production to generate oxidized phospholipids and induced KLF4 expression (Figure 5A). We showed that Abi1‐Δe10 stimulated ROS production, which effect was inhibited by Rac1 and NOX1 inhibition (Figure 5B). Co‐immunoprecipitation assays confirmed that Abi1‐Δe10 promotes active Rac1 interacted with NOX1 in VSMC (Figure 5C). Overexpression of Abi1‐Δe10 increased both KLF4 expression and nuclear localization (Figure 5D‐E). This function of Abi1‐Δe10 could also be attenuated by NOX1 inhibition (Figure 5F‐G). These results revealed a novel action of Abi1‐Δe10 that activates Rac1 and enhance KLF4 function via the Rac1‐NOX1‐ROS axis.

## 4 DISCUSSION

In this manuscript, we report a novel action of Abi1 that contributes to VSMC progression to VSMC‐M. Functionally reprogrammed by the decrease of splicing factor MBNL1, the splice variant of Abi1‐Δe10 is highly expressed in VSMC‐M. Abi1‐Δe10 can interact with Rac1 to trigger the Rac1‐NOX1‐ROS signalling, resulting in the upregulation of KLF4 expression and function, acceleration of VSMC‐M transdifferentiation. These findings highlight the key roles of RNA splicing mechanisms in regulating VSMC phenotype modulation to facilitate atherosclerosis development.

Consensus on the epidemiology of the macrophage‐like cell within the atherosclerotic lesions has not been reached. Multiple hypotheses have been proposed including that this cell originates from blood monocyte‐derived macrophages or VSMC through phenotype modulation.^3^ Accumulating evidence favours the last hypothesis. Lineage‐tracing experiments have demonstrated that VSMC‐derived cells not only form the αSMA‐positive cells but also contribute substantially to the generation of VSMC‐derived cells that lack αSMA but instead express macrophages biomarkers.^4^, ^7^ Although downregulation of MBNL1 expression was reported to be correlated with VSMC proliferation,^21^ we demonstrated for the first time that loss of MBNL1 is a causal event that not only can induce VSMC to express macrophage biomarkers but also can alter cellular functions. We also showed that MBNL1‐associated Abi1‐Δe10 splicing is VSMC‐M specific and highly correlated with macrophage marker status (Figure 1). Abi1‐Δe10 stimulates macrophage biomarkers expression, cell proliferation and macropinocytosis in VSMC‐M (Figure 3). These results highlight the importance of MBNL1 in determining VSMC‐M transdifferentiation and atherosclerosis.

Abi1 is a known adapter protein that has been implicated in actin cytoskeletal remodelling, intercellular adhesion, and smooth muscle migration and contraction upon upstream stimulation.^13^, ^14^, ^16^ However, no study has reported the function difference among Abi1 splicing variants in VSMC‐M. Rac1 and Abi1 are components of the WAVE2 complex that is proposed to regulate VSMC actin polymerization for macropinocytosis,^22^ which probably underlies the critical role of Rac1 in VSMC phenotype alteration. We define a novel action unique to Abi1‐Δe10 (Figure 4 and 5). MBNL1‐mediated RNA splicing of Abi1‐Δe10 promotes Abi1‐Rac1 complex formation in absence of upstream stimulation, resulting in Rac1 membrane localization and activation of the Rac1‐NOX1‐ROS signalling. While it cannot be excluded that the actin remodelling functions of Abi1‐Δe10 may contribute to VSMC‐M development, we demonstrated that both Rac1 and NOX1 inhibition reverse Abi1‐Δe10‐induced VSMC‐M transdifferentiation (Figure 4 and 5), suggesting that the actin remodelling functions of Abi1‐Δe10 may not be essential for VSMC‐M development.

KLF4 is a well‐defined key regulator of cell dedifferentiation. Expression of KLF4 can be induced by oxidized phospholipids.^10^, ^11^ Our results demonstrate that Abi1‐Δe10 engages not only ROS production but also lipid intake through macropinocytosis, both of which promote oxidized phospholipids production. The connection between KLF4 and the macrophage marker expression comes from studies showing that it is a required factor in monocyte differentiation. Thus, it is tempting to speculate that the increased expression of KLF4 after Abi1‐Δe10 overexpression participates not only in the loss of the contractile phenotype but also in the assumption macrophage features in VSMC‐M.

Our study established that loss of MBNL1‐induced Abi1 splicing contributes to VSMC‐M progression. The adapter protein Abi1 is known to mediate various cellular functions triggered by RTKs such as EGFR and PDGFR through downstream effectors such as Rac1.^23^, ^24^, ^25^ What we demonstrate here is that Abi1‐Δe10 bypasses RTKs to activate Rac1 and stimulate downstream signalling. It is now clear that Abi1‐Δe10 enhances the expression and function of KLF4 through the Rac1‐NOX1‐ROS pathway and that KLF4 is key mediators for Abi1‐Δe10 to promote VSMC‐M progression.

## 5 CONCLUSION

Loss‐of‐function of MBNL1 induced Abi1 gene splicing and generation of the Abi1‐Δe10 isoform, which activates VSMC‐M transdifferentiation to promote atherogenesis through Rac1‐NOX1‐ROS‐KLF4 pathway.

## CONFLICT OF INTEREST

The authors declared they do not have anything to disclose regarding conflict of interest with respect to this manuscript.

## AUTHOR CONTRIBUTIONS

Zhang L involved in concept and design. Li Y, Guo X and Xue G involved in acquisition of data. Li Y and Guo X involved in analysis and interpretation of data. Li Y, Ye M, Guo X and Xue G drafted the manuscript. Ye M, Zhao Y and Xue G critically revised the manuscript. Li Y, Ye M, Guo X and Xue G involved in statistical analysis. Li Y, Zhang L and Ni Q obtained funding. Wang H, Wang Y, Wang W, Yang S, Ni Q, Chen J, Lv L, Zhao Y and Xue G involved in administrative, technical or material support.

## ETHICS APPROVAL AND CONSENT TO PARTICIPATE

All procedures were approved by the research ethics committee of Renji Hospital (RA‐2020‐071). All patients included in this research have signed the consents.

## CONSENT FOR PUBLICATION

All authors of the manuscript have read and agreed to its content and are accountable for all aspects of the accuracy and integrity of the manuscript in accordance with ICMJE criteria. The manuscript or portions thereof are not under consideration by any other journal that these findings have not been previously published.

## Supporting information

Supplementary Material

Supplementary Material

## Data Availability

The data that support the findings of this study are available from the corresponding author upon reasonable request.
