# Supplementary material for: RNA Splicing of the Abi1 Gene by MBNL1 contributes to macrophage‐like phenotype modulation of vascular smooth muscle cell during atherogenesis
Source: Cell Prolif. 2021 Mar 23;54(5):e13023. doi: 10.1111/cpr.13023 (PMC8088461; doi:10.1111/cpr.13023)
Supplement: Supplementary file 1 — Supplementary Material [file CPR-54-e13023-s001.docx]

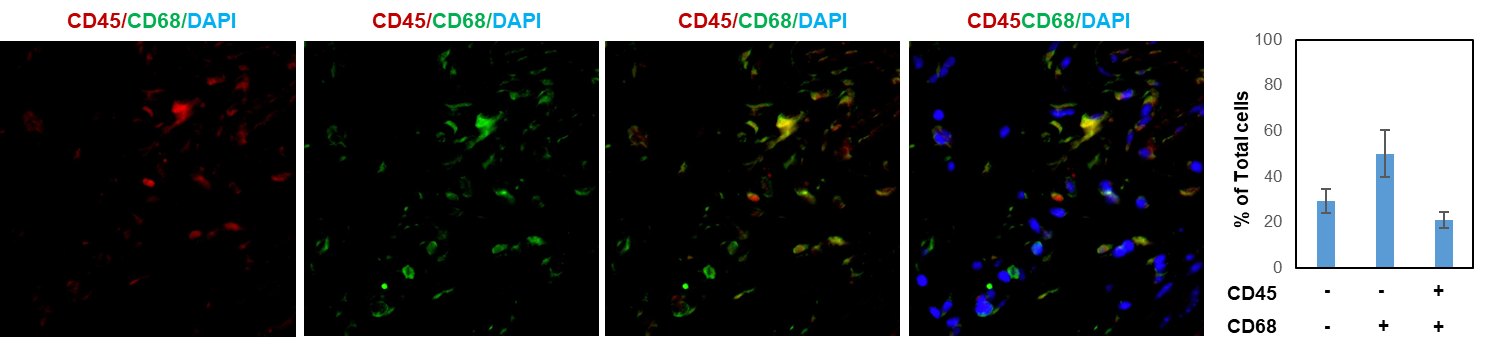


**Supplementary Figure 1**

(**A**) Immunofluorescence staining for CD45 (red, Alexa Fluor 555) and CD68 (green, Alexa Fluor 633) on arteries isolated from patients with severe ASO. Distribution of cell expressing CD45 and/or CD68 were plotted. Representative images of co-staining were shown.


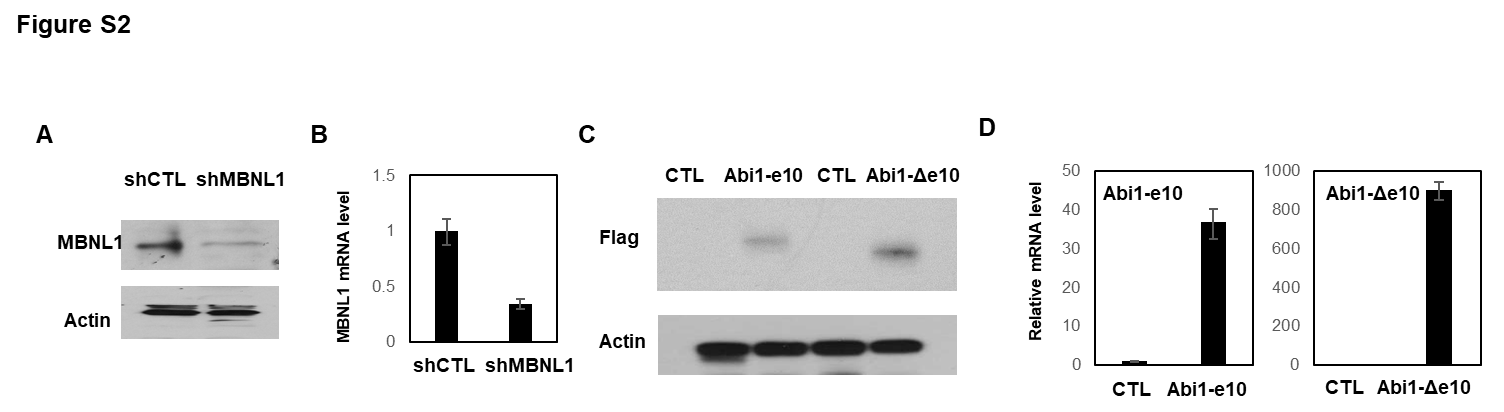


**Supplementary Figure 2**

(**A-B**) VSMC stable cell lines were established by lentivirus expressing control and shMBNL1. After purocymin selection, MBNL1 gene expression was confirmed by real-time qPCR and immunoblotting assays. (**C-D**) VSMC stable cell lines were established by lentivirus expressing control, Abi1-e10 and Abi1-Δe10. After blasticidin selection, Abi1 gene expression was confirmed by real-time qPCR and immunoblotting assays.


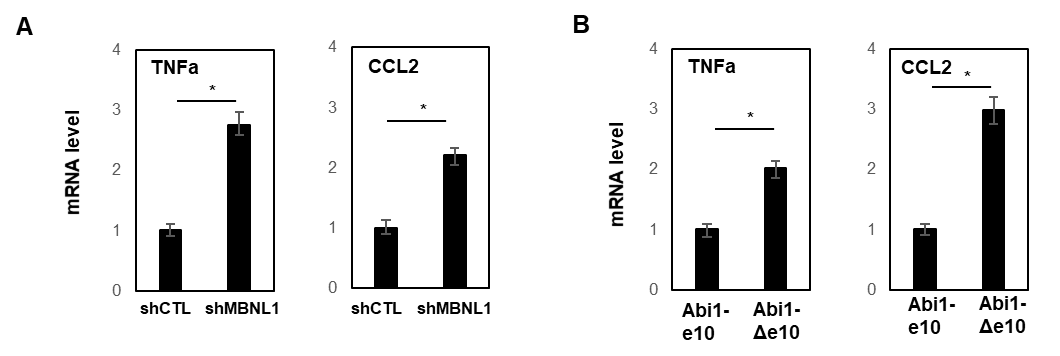


**Supplementary Figure 3**

(**A**) VSMC cell lines were stably transduced by control and shMBNL1 lentivirus, the mRNA and protein expression of TNFα and CCL2 were measured by realtime-qPCR. (**B**)VSMC cell lines were stably transduced by Abi1-e10 and Abi1-Δe10 lentivirus, the mRNA and protein expression of TNFα and CCL2 were measured by realtime-qPCR.

**Supplementary Table1**

**Supplementary Table2**

Primers

| GENE | Sequence |
| --- | --- |
| aSMA F: | GGCATTCACGAGACCACCTAC |
| aSMA R: | CGACATGACGTTGTTGGCATAC |
| 18s rRNA F: | TTGACGGAAGGGCACCACCAG |
| 18s rRNA R: | GCACCACCACCCACGGAATCG |
| AbI1-i F | CATCTTCTGGTGGATACAGACG |
| AbI1-1 R | GGGTGGAGCAATAGAAATTGAA |
| AbI1-2 F | CCACTTTATTCTCAAAATTCAATTGCT |
| AbI1-2 R | GTGGGGGAGGTGGAGAGTC |
| MBNL1 F: | GCTGTTAGTGTCACACCAATTCG |
| MBNL2 R: | AGGCGATTACTCGTCCATTTTC |
| CD68 F: | CTTCTCTCATTCCCCTATGGACA |
| CD68 R: | GAAGGACACATTGTACTCCACC |
| KLF4 F: | CCCACATGAAGCGACTTCCC |
| KLF4 R: | CAGGTCCAGGAGATCGTTGAA |
| RNA-ChIP-ABi1 F: | TGGTCCTTGTAACTGACTTCTGC |
| RNA-ChIP-ABi1 R: | TGTGGAGTCAACTGAGGCATAG |
| RNA-IP-Control F: | TGCACCACCAACTGCTTAGC |
| RNA-IP-Control R: | GGCATGGACTGTGGTCATGAG |

**Supplementary Table3**

Antibodies

| Name | Description | Cat No. | Company |
| --- | --- | --- | --- |
| Actin |  | A2066 | Sigma |
| Flag-tag | M5 | F4042 | Sigma |
| aSMA |  | ab28052 | Abcam |
| MBNL1 |  | ab45899 | Abcam |
| CD68 | FA-11 | ab53444 | Abcam |
| Total Rac1 |  | PA1-091 | Invitrogen |
| B-Spectrin |  | sc-374309 | Santa Cruz |
| Histone H3 |  | 9715 | Cell signaling |
| Tubulin |  | 2144 | Cell signaling |
| KLF4 |  | PA1-095 | Invitrogen |
| NOX1 |  | ab131088 | Invitrogen |
|  |  |  |  |
