# Supplementary material for: RNA Splicing of the Abi1 Gene by MBNL1 contributes to macrophage‐like phenotype modulation of vascular smooth muscle cell during atherogenesis
Source: Cell Prolif. 2021 Mar 23;54(5):e13023. doi: 10.1111/cpr.13023 (PMC8088461; doi:10.1111/cpr.13023)
Supplement: Supplementary file 2 — Supplementary Material [file CPR-54-e13023-s002.docx]

**Supplementary Figure 1**

(**A**) Immunofluorescence staining for CD45 (red, Alexa Fluor 555) and CD68 (green, Alexa Fluor 633) on arteries isolated from patients with severe ASO. Representative images of co-staining were shown.

**Supplementary Figure 2**

(**A-B**) VSMC stable cell lines were established by lentivirus expressing control, Abi1-e10, and Abi1-Δe10. After blasticidin selection, Abi1 gene expression was confirmed by real-time qPCR and immunoblotting assays. (**C-D**) VSMC stable cell lines were established by lentivirus expressing control and shMBNL1. After puromycin selection, MBNL1 gene expression was confirmed by real-time qPCR and immunoblotting assays.

**Supplementary Figure 3**

(**A**) VSMC cell lines were stably transduced by control and shMBNL1 lentivirus, the mRNA and protein expression of TNFα and CCL2 were measured by realtime-qPCR. (**B**)VSMC cell lines were stably transduced by Abi1-e10 and Abi1-Δe10 lentivirus, the mRNA and protein expression of TNFα and CCL2 were measured by realtime-qPCR.

**Supplementary table1**

Demographic information of patients

**Supplementary table2**

Information of realtime-qPCR primers used in this manuscript

**Supplementary table3**

Information of antibodies used in this manuscript
